# Correlation analysis and gender differences of cognitive function based on mini-mental state examination (MMSE) and suicidal tendency in patients with schizophrenia

**DOI:** 10.1186/s12888-023-05462-9

**Published:** 2024-01-02

**Authors:** Quanfeng Zhu, Xiang-Yang Zhang

**Affiliations:** 1 Affiliated Xiaoshan Hospital, Hangzhou Normal University, 728 Yucai North Road, Hangzhou, 311201 China; 2 CAS Key Laboratory of Mental Health, Institute of Psychology, 16 Lincui Rd, Beijing, 100101 China; 3 Department of Psychology, University of Chinese Academy of Sciences, 16 Lincui Rd, Beijing, 100101 China

**Keywords:** Gender differences, Schizophrenia, Cognitive dysfunction, Suicidal tendency, Mini-mental state examination

## Abstract

**Background:**

The aim of this study was to investigate the correlation and gender differences between cognition and suicidal tendency in patients with schizophrenia.

**Methods:**

A total of 554 patients with schizophrenia were recruited. The Mini-Mental State Examination (MMSE), Positive and Negative Syndrome Scale (PANSS), Interpersonal Reactivity Index (IRI), Toronto Alexithymia Scale (TAS), and Insomnia Severity Index (ISI) were used to assess clinical symptoms.

**Results:**

In male patients, MMSE score and the incidence of suicidal tendency were correlated (P = 0.04, OR = 1.06, 95%CI: 1.00–1.12). Among patients with cognitive dysfunction, IRI score (P = 0.01, OR = 1.04, 95%CI: 1.01–1.06), and types of antipsychotic drugs (P < 0.01, OR = 3.97, 95%CI: 1.76–8.97) in male patients were associated risk factors for suicidal ideation. Among patients without cognitive dysfunction, PANSS positive subscale score (P = 0.03, OR = 1.06, 95%CI: 1.01–1.11), and PANSS general psychopathology score (P = 0.02, OR = 1.05, 95%CI: 1.01–1.08) were associated risk factors for suicidal ideation in male patients and PANSS positive subscale score (P < 0.01, OR = 1.15, 95%CI: 1.05–1.26) were associated risk factors for suicidal ideation in female patients.

**Conclusions:**

There were significant gender differences in the correlation between cognitive functioning and suicidal ideation in patients with schizophrenia. Cognitive function may play an important mediating role in other factors on suicide.

## Introduction

Schizophrenia is a psychiatric disorder with severe and complex symptoms. People with schizophrenia experience hallucinations and cognitive impairment, therefore, impairment of consciousness pathways may be a central feature of schizophrenia [1]. The prevalence of schizophrenia is high, with an average lifetime prevalence of about 1%, but varies widely across regions and populations [2]. Schizophrenia poses a serious threat to social stability, as it can largely impair the social functioning of patients and disconnect them from society [3]. In addition, the stigma of schizophrenia can be extremely damaging to individuals, families, and society [4]. Schizophrenia is a difficult disorder to treat and its pathogenesis is still not well studied. However, studies in recent decades have shown that genetic and prenatal factors play a key role in the pathogenesis of schizophrenia [5]. On this basis, risk models based on genetic and environmental interactions have been proposed to differentiate schizophrenia outcomes [6].

There are significant gender differences in schizophrenia, more commonly in age of onset, hormonal involvement, and general brain abnormalities, which are, of not specific to schizophrenia [7]. A study based on a Chinese population sample showed that women with schizophrenia had more severe difficulties with understanding and communication, whereas men had more severe dysfunction in daily activities [8]. In addition, a study by Abel et al. revealed the same gender differences in response to antipsychotic medications, which may be related to complex gender-specific endocrine effects [9].

Cognitive impairment is common in patients with schizophrenia [10]. A growing number of studies have shown that schizophrenia can cause significant impairments in neurological and social cognitive functions such as episodic memory, executive function, social cognition, complex cognition, and sensorimotor functions [11]. In addition, people with schizophrenia have been found to have impairments in face emotion recognition, as well as empathic and narrative functioning. Several previous studies have found that these dysfunctions in schizophrenic patients correlate with the severity of psychotic symptoms and negative symptoms [12, 13]. Impairment of these functions severely affects the daily social interaction of patients, and disconnects them from society. Meanwhile, some studies have also pointed out that cognitive impairment in patients with schizophrenia may be associated with suicide. For example, Yin et al. showed that among patients with first-episode schizophrenia, those with more severe impairment in specific domains of neurocognitive functioning had a higher probability of suicide attempts [14]. However, a study by Delaney et al. had different results, they found that patients with suicide attempts had better cognitive functioning than those without suicide attempts [15].

Suicide is the leading cause of death in patients with schizophrenia. Compared with the healthy population, patients with schizophrenia have a significantly higher rate of suicide [16]. Several studies have confirmed that lifetime suicide rates of patients with schizophrenia reach approximately 10%, and factors such as younger age, being male, unmarried, living alone, occupation, and higher literacy levels are associated with potentially higher rates of suicide [17]. Similarly, a study by Balhara’s team confirmed that being male and unmarried were associated with an increased risk of suicide in patients with schizophrenia [18].

Schizophrenia patients often co-morbidly suffer from insomnia, and a number of studies have found significant abnormalities in sleep structure and sleep oscillatory rhythms in schizophrenia, and sleep disorders have even been suggested to be one of the most important factors in the development of psychiatric disorders [19, 20]. Studies have shown that sleep disorders are strongly associated with suicide risk and may even be a predictor of suicide [21]. Sleep disorders are also associated with suicidal ideation and suicide attempts in patients with schizophrenia [22].

In addition, our previous study confirmed that positive symptoms, negative symptoms, empathy and other factors are associated with suicide attempts in patients with schizophrenia, and that positive symptoms and empathy are risk factors for suicide attempts [23]. However, there are no reports on whether there are gender differences in the association of these factors with suicide attempts in schizophrenia. Just as there are prevalent sex differences in many aspects of schizophrenia, we propose that there are also sex differences in the factors mentioned above. The aim of this study was to explore the gender differences in association between cognitive impairment and suicide tendency in patients with schizophrenia, and the gender differences in influence of positive symptoms, negative symptoms, sleep disorders, empathy and other factors on suicide tendency in patients with different cognitive abilities.

## Methods

### Subjects

The authors assert that all procedures contributing to this work comply with the ethical standards of the relevant national and institutional committees on human experimentation and with the Helsinki Declaration of 1975, as revised in 2008. All procedures involving human subjects/patients were approved by the Ethics Committee of Institute of Psychology, Chinese Academy of Sciences (approval number: H18031), and each participating subject had signed a written informed consent form.

The present study was a cross-sectional study. A total of 554 inpatients with schizophrenia from Guangzhou Huiai Hospital and Wuhan Xinzhou Mental Health Center were recruited. Our recruitment criteria were as follows: (1) diagnosed with schizophrenia after independent assessment by two trained psychiatrists using the Structured Clinical Interview for DSM-IV (SCID); (2) Han Chinese population aged 18–70 years; (3) stable doses of antipsychotic drugs were taken for more than six months at the time of recruitment; and (4) were able to sign written informed consent and cooperate with scale assessment. And the exclusion criteria were: (1) comorbid with serious somatic or autoimmune diseases; (2) comorbid with mental retardation or severe neurological disorders; (3) were unable to cooperate with the scale assessment due to the fluctuations of psychotic symptoms; and (4) pregnant or lactating women.

### Assessment of general information

Information on the age, gender, years of education, marital status, family history, history of diabetes and history of hypertension was collected from each patient through questionnaires. All the above information was investigated with guardians or family members to ensure the accuracy. In addition, the height and weight of each patient were measured. Body mass index (BMI) of each patient was calculated by dividing the weight in kilograms by the height in meters squared. Moreover, we investigated the subjects’ medication use and recorded the types of antipsychotic drugs taken by each subject and the total dose after converting to chlorpromazine equivalent.

### Clinical interview and assessment

In this study, clinical symptoms of subjects were assessed by two professional psychiatrists using the following scales. The two psychiatrists were trained in the use of each scale prior to scale assessment. After training, the correlation coefficient of each scale between the two psychiatrists was above 0.8. The subjects included in this study were hospitalized patients, and all scale assessments were completed in the ward. To avoid fatigue, each subject was assessed on one scale by two psychiatrists separately each day. In addition, to avoid phenomenon of scoring by impression, each subject was assessed by two different scales each day.

#### Assessment of psychiatric symptoms

The Positive and Negative Syndrome Scale (PANSS) was used to assess subjects’ psychiatric symptoms. It contains three subscales of positive symptom, negative symptom, and general psychopathology. The positive and negative symptom subscales contain 7 items each, and the general psychopathology subscale contains 16 items, each of which is scored from 1 to 7 on a scale of severity, with higher scores indicating more severe symptoms. The Chinese version of PANSS has been confirmed to have good reliability in Chinese patients with schizophrenia in remission (Cronbach’s α coefficient = 0.93) [24].

#### Assessment of suicidal tendency and intensity of suicidal ideation

Suicide ideation is defined as having suicidal thoughts, but not carrying out suicidal actions, whereas suicide attempts are defined as carrying out suicidal actions [25]. Each patient was asked questions about suicide in the form of a face-to-face interview, such as “Have you ever had suicidal thoughts” and “Have you ever attempted suicide in your life?” If so, further data were collected on the time, method and exact date of each suicide attempt. All information collected was verified with guardians or family members. Patients were then classified into the following three categories: (1) no suicidal ideation; (2) suicidal ideation but no suicide attempts; and (3) suicide attempts. In this study, patients who met (1) were considered to have no suicidal tendency, and those who met (2) or (3) were considered to have suicidal tendency. In addition, the Beck Scale for Suicide Ideation-Chinese Version (BSI-CV) was used to assess suicidal ideation and suicidality in subjects. BSI-CV contains 19 items, each of which is asked about two time periods: the most recent week and the most depressed. The scale has been proved to have good reliability, validity and test-retest consistency in Chinese population [26].

#### Assessment of the severity of insomnia

The Insomnia Severity Index (ISI) was used to assess the severity of insomnia in each subject. ISI contains 7 items with a total of 28 points, each item is scored from 0 to 4, with higher scores indicating more severe insomnia. ISI is widely used to assess the severity of insomnia, and its effectiveness has also been verified in patients with schizophrenia [27]. The Chinese version of ISI has been proved to have high reliability (Cronbach’s α coefficient = 0.81) [28].

#### Assessment of cognitive function

Based on the wide range of applications of Mini-Mental State Examination (MMSE) in the field of psychiatry, it was used to perform a simple screening of patients for cognitive function in this study. The scale of MMSE was scored out of 30, with higher scores representing better cognitive function. The MMSE is less accurate for screening cognitive impairment in the elderly, and in addition, education level can have a large impact on MMSE scores [29]. In the present study, we excluded elderly people over 70 years of age and corrected for educational attainment using screening criteria commonly used in the Chinese region: cutoff value for the 0–4 years of education group: ≤19; cutoff value for the 5–8 years of education group: ≤22; cutoff value for the > 8 years of education group: ≤26 [30]. The sensitivity, specificity and accuracy of the optimized Chinese version of MMSE were all higher than 90% [30].

#### Assessment of empathy and alexithymia

The Interpersonal Reactivity Index (IRI) and the Toronto Alexithymia Scale (TAS) were used to assess patients’ empathy and narrative abilities, respectively.

IRI included 4 subscales, each containing 7 items, and each item was scored from 1 to 5 according to the degree of compliance. Two of the subscales were used to assess subjects’ cognitive empathy: Perspective Taking (PT) and fantasy (FS), and the other two were used to assess emotional empathy: Empathic Concern (EC) and Personal Distress (PD). Patients with higher IRI scores are more empathic. IRI is widely used to assess empathy in patients with schizophrenia [31]. In addition, the Chinese version of the scale has been confirmed to have good construct reliability, test-retest reliability and construct validity [32].

TAS consists of 20 items and contains 3 factors: F1: emotion discrimination, F2: emotion description, and F3: extroverted thinking. Each item was scored from 1 to 5 according to the degree of coincidence, and the total score ranged from 20 to 100, with higher scores indicating more severe alexithymia. The Cronbach’s α coefficient of the Chinese version of TAS were 0.84, and the test-retest reliability was 0.87 [33].

### Data analysis

The Shapiro-Wilk Test was used to perform normality tests. Analysis of Variance (ANOVA) was applied for continuous variables that conformed to a normal distribution, Mann-Whitney U Test for continuous and rank variables that did not conform to a normal distribution, and Chi-Square Test for categorical variables to compare sociodemographic and clinical variables for male and female patients, respectively. Binary logistic regression analysis was utilized to calculate odds ratio (OR) for suicidal tendency for male and female patients separately.

Data that conformed to a normal distribution were expressed as mean ± standard deviation, and data that did not conform to normal distribution were expressed as median (25th quartile to 75th quartile). All statistical analyses for this study were completed on SPSS 25.0. The results of all Univariate analyses in this study were corrected by false discovery rate (FDR), and the significance threshold for corrected q-values was set at 0.05.

## Results

### Gender differences in the prevalence of suicidal tendency in patents with and without cognitive dysfunction

The prevalence of cognitive dysfunction in male and female patients with schizophrenia was 38.3% (145/379) and 44.6% (78/175), respectively. And the prevalence of suicidal tendency in male and female patients with cognitive dysfunction was 22.8% (33/145) and 29.5% (23/78), respectively. And the prevalence of suicidal tendency in male and female patients without cognitive dysfunction was 32.5% (76/234) and 36.1% (35/97), respectively. As shown in Table 1, compared to patients without cognitive dysfunction, both male and female patients with cognitive dysfunction were older, had longer duration of disorder, and higher PANSS scores (all q values < 0.05). In addition, compared with patients without cognitive dysfunction, male patients with cognitive dysfunction had higher TAS, and ISI scores.

**Table 1:** Sociodemographic and clinical characteristics of male and female patients with and without cognitive dysfunction

Variable	Male				Female			
	Patients with cognitive dysfunction (N = 145)	Patients without cognitive dysfunction (N = 234)	F/χ2/Z	q value	Patients with cognitive dysfunction (N = 78)	Patients without cognitive dysfunction (N = 97)	F/χ2/Z	q value
Age	52(42.5 ~ 59)	45(34 ~ 54)	-4.70	< 0.001	47(38 ~ 56.25)	41(31.5 ~ 50)	-3.27	< 0.01
Duration of disorder, years	25(16 ~ 32.5)	19(10 ~ 29)	-3.71	< 0.001	21(11.75 ~ 33)	14(10.5 ~ 22.5)	-3.37	< 0.01
Years of education	9(8 ~ 11)	8(8 ~ 11)	-1.18	0.33	8(6.75 ~ 11)	8(8 ~ 12)	-1.10	0.47
BMI, kg/m2	24.24(21.30 ~ 26.20)	24.23(21.63 ~ 26.81)	-0.53	0.66	24.65(21.88 ~ 27.54)	25.15(22.23 ~ 28.49)	-0.79	0.59
Total dose of antipsychotic medication, mg	280(150 ~ 400)	250(150 ~ 360)	-1.21	0.32	225(120 ~ 385)	200(105 ~ 350)	-0.81	0.59
Types of antipsychotic drugs	1(1 ~ 2)	1(1 ~ 2)	-0.23	0.82	2(1 ~ 2)	2(1 ~ 2)	-0.06	0.95
PANSS positive subscale score	16(13 ~ 19)	14(12 ~ 18)	-2.43	0.03	18(14 ~ 22)	16(12 ~ 19)	-2.85	0.01
PANSS negative subscale score	23(18 ~ 27.5)	19(16 ~ 22)	-5.46	< 0.001	24(20.75 ~ 30)	18(15 ~ 24)	-5.10	< 0.001
PANSS general psychopathology subscale score	42(37 ~ 45.5)	37(32 ~ 43)	-5.37	< 0.001	44.35 ± 9.18	38.30 ± 9.07	0.32	< 0.001
PANSS total score	81(72 ~ 90)	71(62 ~ 81)	-5.93	< 0.001	87.5(74.5 ~ 102.25)	73(61 ~ 86)	-4.94	< 0.001
IRI score	84(71 ~ 96)	87(78 ~ 96)	-1.45	0.22	86.55 ± 13.55	88.95 ± 14.27	0.79	0.47
PT perspective taking	21(17 ~ 25)	23(19 ~ 26)	-2.88	< 0.01	21.97 ± 5.34	22.88 ± 5.09	0.01	0.47
FS fantasy	19.82 ± 6.57	20.10 ± 5.28	11.30	0.71	19(15 ~ 25)	20(17 ~ 26)	-1.37	0.35
EC empathic concern	23(18.5 ~ 25)	23(19 ~ 26)	-1.62	0.17	23.5(20 ~ 26.25)	24(20 ~ 27)	-0.44	0.72
PD personal distress	21(17 ~ 24)	21(18 ~ 24)	-0.29	0.79	22(18 ~ 25)	22(18 ~ 25.5)	-0.29	0.80
TAS score	58.99 ± 9.67	55.79 ± 8.99	0.31	< 0.01	55.27 ± 8.59	54.29 ± 10.68	2.57	0.63
F1 emotion discrimination	21(17 ~ 25)	19(15 ~ 23)	-2.29	0.04	17(15 ~ 23)	17(14.5 ~ 22)	-0.29	0.80
F2 emotion description	15(12 ~ 18)	14(12 ~ 17)	-0.86	0.46	13.95 ± 3.81	14.78 ± 3.79	0.02	0.33
F3 extraversion thinking	23(21 ~ 26)	22(20 ~ 24)	-3.30	< 0.01	22(20 ~ 26)	21(18 ~ 24)	-2.73	0.02
FERS score	27(20 ~ 33)	32(27 ~ 37)	-6.05	< 0.001	23.95 ± 8.63	32.48 ± 7.29	0.84	< 0.001
Fear	5(4 ~ 7)	6(4 ~ 10)	-4.00	< 0.001	4(1.75 ~ 7)	6(4 ~ 9)	-3.31	< 0.01
Anger	6(4 ~ 8)	7(5 ~ 10)	-3.63	< 0.001	5.5(3 ~ 8)	7(5 ~ 9)	-3.90	< 0.001
Joy	6(4 ~ 9)	7(5 ~ 9)	-3.44	< 0.01	6.5(4 ~ 10)	8(6 ~ 10.5)	-1.77	0.18
Neutrality	9(6 ~ 12)	11(9 ~ 13)	-4.03	< 0.001	8(2 ~ 11)	10(9 ~ 13)	-5.23	< 0.001
ISI score	2(1 ~ 4)	1(0 ~ 4)	-2.30	0.04	2(1 ~ 5)	1(0 ~ 4)	-1.90	0.15
BSI score	10(10 ~ 12)	10(10 ~ 41)	-2.00	0.08	10(10 ~ 14.5)	10(10 ~ 29)	-0.576	0.66
Suicidal tendency			4.13	0.08			0.85	0.56
Yes, n (%)	33(22.8%)	76(32.5%)			23(29.5%)	35(36.1%)		
No, n (%)	112(77.2%)	158(67.5%)			55(70.5%)	62(63.9%)		
Diabetes			0.34	0.64			1.04	0.51
Yes, n (%)	19(13.1%)	26(11.1%)			13(16.7%)	11(11.3%)		
No, n (%)	126(86.9%)	208(88.9%)			65(83.3%)	86(88.7%)		
Hypertension			1.17	0.34			0.65	0.59
Yes, n (%)	23(15.9%)	28(12.0%)			13(16.7%)	12(12.4%)		
No, n (%)	122(84.1%)	206(88.0%)			65(83.3%)	85(87.6%)		
Family history			1.32	0.33			0.51	0.60
Yes, n (%)	13(9.0%)	30(12.8%)			18(23.1%)	27(27.8%)		
No, n (%)	132(91.0%)	204(87.2%)			60(76.9%)	70(72.2%)		
Smoking history			-1.09	0.34			-0.55	0.66
Never, n (%)	61(42.1%)	82(35.0%)			74(94.9%)	90(92.8%)		
Past smoking, n (%)	24(16.5%)	47(20.1%)			3(3.8%)	6(6.2%)		
Current smoking, n (%)	60(41.4%)	105(44.9%)			1(1.3%)	1(1.0%)		
Marital status			5.70	0.20			2.66	0.59
Unmarried, n (%)	97(66.9%)	171(73.0%)			27(34.6%)	42(43.3%)		
Married, n (%)	26(17.9%)	28(12.0%)			26(33.4%)	34(35.0%)		
Divorced, n (%)	22(15.2%)	32(13.7%)			21(26.9%)	18(18.6%)		
Widowed, n (%)	0	3(1.3%)			4(5.1%)	3(3.1%)		

Furthermore, after including age and duration of disorder as covariates, binary logistic regression analysis showed that suicidal tendency was significantly associated with MMSE score (P = 0.04, OR = 1.06, 95%CI: 1.00–1.12) in male patients. However, in female patients, MMSE scores and suicidal tendency were not correlated (P > 0.05).

### Gender differences in indicators between suicidal and non-suicidal patients with/without cognitive dysfunction

As shown in Tables 2 and 3, among patients with cognitive dysfunction, BSI scores were higher in male and female patients with suicidal tendency compared with those without suicidal tendency (both q values < 0.001). In addition, male patients with suicidal tendency had higher IRI scores and took more types of antipsychotic drugs than those male patients without suicidal tendency (both q values < 0.05). And among patients without cognitive dysfunction, BSI and PANSS positive subscale scores were higher in male and female patients with suicidal tendency compared with those without suicidal tendency (all q values < 0.05). Besides, male patients with suicidal tendency had higher PANSS general psychopathology scores (q value < 0.05).

**Table 2:** Sociodemographic and clinical characteristics of male and female patients with cognitive dysfunction

Variable	Male				Female			
	Patients with suicidal tendency (N = 33)	Patients without suicidal tendency (N = 112)	F/χ2/Z	q value	Patients with suicidal tendency (N = 23)	Patients without suicidal tendency (N = 55)	F/χ2/Z	q value
IRI score	90.03 ± 13.65	80.55 ± 18.13	4.39	0.02	87(80 ~ 103)	84(75 ~ 94)	-1.11	0.71
BSI score	51(39 ~ 62)	10(10 ~ 10)	-9.67	< 0.001	57(37 ~ 67)	10(10 ~ 10)	-7.61	< 0.001
Types of antipsychotic drugs	2(1 ~ 2)	1(1 ~ 2)	-3.36	0.02	2(1 ~ 2)	2(1 ~ 2)	-0.32	0.89

**Table 3:** Sociodemographic and clinical characteristics of male and female patients without cognitive dysfunction

Variable	Male				Female			
	Patients with suicidal tendency (N = 76)	Patients without suicidal tendency (N = 158)	F/χ2/Z	q value	Patients with suicidal tendency (N = 35)	Patients without suicidal tendency (N = 62)	F/χ2/Z	q value
PANSS positive subscale score	15(14 ~ 18.75)	14(11 ~ 17)	-3.02	0.03	18(15 ~ 22)	14(12 ~ 18)	-3.06	0.03
PANSS general psychopathology subscale score	40.5(33.25 ~ 44.75)	36(32 ~ 41)	-3.15	0.03	43(34 ~ 45)	36.5(31 ~ 42.25)	-1.78	0.71
BSI score	47(22.5 ~ 52.75)	10(10 ~ 10)	-10.74	< 0.001	49(14 ~ 61)	10(10 ~ 10)	-7.71	< 0.001

### Gender differences in risk factors associated with suicidal tendency in patients with/without cognitive dysfunction

To explore the degree of influence of the above significantly different factors on suicidal tendency, we performed binary logistic regression analysis with suicidal tendency as the dependent variable. In male patients with cognitive dysfunction, IRI score (P = 0.01, OR = 1.04, 95%CI: 1.01–1.06), and types of antipsychotic drugs (P < 0.01, OR = 3.97, 95%CI: 1.76–8.97) were associated risk factors for suicidal tendency. In male patients without cognitive dysfunction, BSI score (P < 0.01, OR = 1.12, 95%CI: 1.09–1.15), PANSS positive subscale score (P = 0.03, OR = 1.06, 95%CI: 1.01–1.11), and PANSS general psychopathology score (P = 0.02, OR = 1.05, 95%CI: 1.01–1.08) were associated risk factors for suicidal tendency. In female patients without cognitive dysfunction, the risk factors associated with suicidal tendency were BSI score (P < 0.01, OR = 1.13, 95%CI: 1.06–1.21), and PANSS positive subscale score (P < 0.01, OR = 1.15, 95%CI: 1.05–1.26).

## Discussion

To our knowledge, this study is the first to compare gender differences in the correlation between cognitive functioning and suicidal tendency in patients with schizophrenia. Many previous studies have reported correlations between suicidal ideation and suicide attempts and cognitive functioning in patients with schizophrenia, but the results have been inconsistent. For example, the results of Delaney et al. showed that patients with suicidal ideation or attempts had better cognitive functioning overall than those who did not have suicidal ideation [15]. In contrast, Yin’s team found that patients with more impaired cognitive functioning had a higher probability of suicide attempts [14]. However, a study by Zoghbi and Dai et al. found no significant association between suicide attempts and cognitive functioning in patients with schizophrenia [34]. The heterogeneity of the samples may have contributed to these different findings. Despite the inconsistent findings, the previous research views prefer that poorer cognitive functioning provides some protection against suicidal ideation [35]. However, no gender differences in the association between cognitive function and suicide have been reported. In the present study, the association between cognitive impairment based on optimized MMSE and suicidal tendency was significant in male patients but not in female patients. After adjustment for P value, the association of cognitive impairment with suicidal tendency was also no longer significant in male patients. However, after excluding the interference of age and disease duration, MMSE score was a risk factor for suicidal tendency in male patients, but not in female patients. We suggest that cognitive impairment may influence suicide by reducing the ability to plan and carry out suicide attempts. We hypothesize that gender differences in self-control may contribute to the gender differences in the correlation between cognition and suicidal tendency in schizophrenic patients. The stronger self-control in females may have weakened the correlation between cognitive functioning and suicidal tendency [36]. Whether this gender difference in the association between cognitive function and suicidal tendency in schizophrenia is widespread or simply due to sample heterogeneity remains to be further investigated with a larger sample. Another interesting finding of this study was that male patients with cognitive impairment, but not female patients with cognitive impairment, were accompanied by a significant decline in narrative ability, which seems to indicate that the expressive ability of female patients with schizophrenia is less affected by cognitive impairment.

We found that in both male and female, positive symptoms were associated with suicidal tendency only among those without cognitive impairment. Some previous studies have also found that more severe positive symptoms were significantly associated with suicide attempts in patients with chronic schizophrenia [37]. Positive symptoms may influence suicide in a number of ways, directly or indirectly, and guilt delusions are thought to be most strongly associated with increased risk of suicidality, a belief that may exacerbate suicidal thoughts [38]. And the correlation between fantasy and suicide has a similar explanation, especially as hallucinations that are composed of suicidal needs directly drive suicidal behavior [39]. Our findings revealed that cognition may mediate the association between positive symptoms and suicidal tendency, because although positive symptom was more severe in patients with cognitive impairment, positive symptom was a risk factor for suicidal tendency only in patients without cognitive impairment.

In the present study, the association between empathy and suicidal tendency was significant only in male patients with cognitive impairment. However, excluding the effects of gender and cognition, personal distress consistently had the strongest association with suicidal tendency. Patients with more intense personal distress experience more intense anxiety and discomfort in daily communication, which may be responsible for the increased risk of suicide. However, our findings suggested that the association between empathy and suicidal tendency may also be modulated by cognition. In addition, the reasons for gender differences in the associations of empathy and suicidality still need further investigation.

Our study revealed that among patients with cognitive dysfunction, suicidal tendency in male was associated with greater variety of antipsychotic drug taken. First, more medication use itself is associated with more severe conditions. Second, there are gender differences in cognitive impairment of patients with some antipsychotic medications, which may have contributed to the gender differences in our study.

Our study also has some limitations. First, the present study was a cross-sectional study, which could not explain the causal relationship between cognitive functioning, suicidal tendency, and other factors. Second, the population recruited for this study was a Chinese Han population. Culture and customs in different countries and regions have a strong influence on suicidality, so the findings of this study may not be applicable to other countries and regions. Third, because we collected insufficient biochemical indicators, this study lacks an exploration of the correlation between cognitive function, suicidal ideation, and biochemical indicators.

In conclusion, this study explored the correlation between cognition and suicidal tendency in patients with schizophrenia as well as gender differences. We also identified a possible mediating role of cognitive function in other factors influencing suicidal ideation. Next, we will continue to explore the effects of different types of positive symptoms, empathy and other factors on suicidal tendency and the way through which cognition plays a mediating role between these factors and suicidal tendency.

## Data Availability

The data that support the findings of this study are available from the corresponding author upon reasonable request.
